# Visual imagination can influence visual perception – towards an experimental paradigm to measure imagination

**DOI:** 10.1038/s41598-024-74693-x

**Published:** 2024-10-18

**Authors:** Azadeh Mozhdehfarahbakhsh, Lukas Hecker, Ellen Joos, Jürgen Kornmeier

**Affiliations:** 1 Institute for Frontier Areas of Psychology and Mental Health, Freiburg, Germany; 2 Faculty for Biology, University of Freiburg, Freiburg im Breisgau, Germany; 3 Department of Psychiatry and Psychotherapy, Medical Center - University of Freiburg, Freiburg im Breisgau, Germany; 4 Faculty of Medicine, University of Freiburg, Freiburg im Breisgau, Germany

**Keywords:** Neuroscience, Psychology

## Abstract

During visual imagination, a perceptual representation is activated in the absence of sensory input. This is sometimes described as seeing with the mind’s eye. A number of physiological studies indicate that the brain uses more or less the same neural resources for visual perception of sensory information and visual imagination. The intensity of visual imagination is typically assessed with questionnaires, while more objective measures are missing. Aim of the present study was, to test a new experimental paradigm that may allow to objectively quantify imagination. For this, we used priming and adaptation effects during observation of ambiguous figures. Our perception of an ambiguous stimulus is unstable and alternates spontaneously between two possible interpretations. If we first observe an unambiguous stimulus variant (the conditioning stimulus), the subsequently presented ambiguous stimulus can either be perceived in the same way as the test stimulus (priming effect) or in the opposite way (adaptation effect) as a function of the conditioning time. We tested for these conditioning effects (priming and adaptation) using an ambiguous Necker Cube and an ambiguous Letter /Number stimulus as test stimuli and unambiguous variants thereof as conditioning stimuli. In a second experimental condition, we tested whether the previous imagination of an unambiguous conditioning stimulus variant – instead of its observation – can have similar conditioning effects on the subsequent test stimulus. We found no systematic conditioning effect on the group level, neither for the two stimulus types (Necker Cube stimuli and Letter /Number stimuli) nor for the two conditions (Real and Imaginary). However, significant correlations between effects of Real and Imaginary Condition were observed for both stimulus types. The absence of conditioning effects at the group level may be explained by using only one conditioning time, which may fit with individual priming and adaptation constants of some of our participants but not of others. Our strong correlation results indicate that observers with clear conditioning effects have about the same type (priming or adaptation) and intensity of imaginary conditioning effects. As a consequence, not only past perceptual experiences but also past imaginations can influence our current percepts. This is further confirmation that the mechanisms underlying perception and imagination are similar. Our post-hoc qualitative observations from three self-defined aphantasic observers indicate that our paradigm may be a promising objective measure to identify aphantasia.

**Supplementary Information:**

The online version contains supplementary material available at 10.1038/s41598-024-74693-x.

## Introduction

### Perception without sensory input

Visual imagination can be defined as the activation of a perceptual representation in the absence of sensory input^1^. It is sometimes also described as “seeing with the mind’s eye”. Research has provided a growing body of behavioral and neuroimaging evidence indicating a considerable overlap between the mechanisms underlying imagination and perception^2–5^. For example, both perception and visual imagination are affected when visual cortical activity is impaired by transcranial magnetic stimulation^6^, indicating that the visual cortex is involved in imagination. Moreover, recent neuroimaging studies illustrate that there is remarkable spatial overlap between the sensory and higher-level brain areas when they are activated during visual perception of an object and during its visual imagination^6–10^(for recent reviews see^11,12^). Visual perception is regarded as an interplay between bottom-up sensory evidence and top-down expectations and perceptual memory contents (e.g^13,14^). Given the above described findings, imagination can be regarded as a more or less voluntarily induced extreme case of perception where the bottom-up source of information is absent and perception is purely based on top-down information.

A type of deviation from normal imagination has been introduced by Zeman et al^15,16^. The authors coined the term “aphantasia” to describe individuals who are congenitally unable to evoke vivid depictive visual imagination, but instead use more symbolic, propositional or phonological imaginations. Extreme aphantasia is currently estimated to have a prevalence of 0.7% ^17^. The authors further introduced the term “hyperphantasia” in order to describe a subpopulation of humans with the ability to evoke imaginations “as vivid as real seeing”^15,18^. Extreme hyperphantasia is estimated to have a prevalence of 2.5%. Another type of deviation from normal imagination is the phenomenon of “eidetic memory”, sometimes also labelled “photographic memory”. It relates to the ability of vivid imaginations after only few instances of exposure^19^. People with eidetic memory can visualize tiny details of scenes and objects, as if they have them in front of their eyes. Eidetic memory is a rare and controversially discussed phenomenon^20^. Typical imagination questionnaires, like the VVIQ (Vividness of Visual Imagery Questionnaire^21^), had been designed to reflect imagination abilities in the general population.

It is currently unclear how much and in which way the visual system is involved in imagination of aphantasics and what the major neurophysiological differences are to visual imagination in regular imaginers. Aphantasia may thus be a conceptually different way of imagination rather than an extreme on a continuous scale, which makes the usability of standard imagery questionnaires problematic. Given the variety of imagination phenomena and intensities, it may be helpful to have an objective measure at hand – beyond typical questionnaires –that allows to investigate, whether a person has at all the ability of visual imagination, and if yes, to quantify the intensity of this imagination ability. In the following, we propose an experimental paradigm, using ambiguous figures, that may serve this purpose.

### Conditioning effects with ambiguous figures

**Fig. 1:**
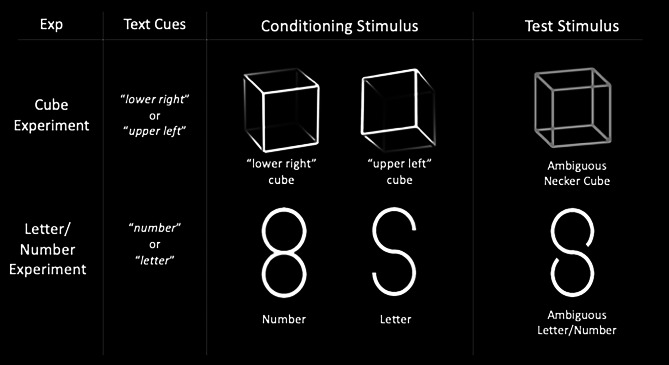
Stimuli and Cueing Texts. The ‘Exp’ column indicates the Necker Cube and Letter/Number experiments. The ‘Text Cues’ column displays the text cues that were shown as prompts in both the Real Condition and Imaginary Condition before presenting the stimuli. The ‘Conditioning Stimulus’ column displays the unambiguous conditioning stimulus variants along with their corresponding text labels. The ‘Test Stimuli’ column presents the ambiguous test stimuli from both experiments for which participants were required to respond. The labels below the stimuli are shown here for explanation purposes, but were not displayed during the experiment.

Our everyday experience suggests that our percepts represent the world as it is. However, the (bottom-up) information available via our senses is incomplete, noisy, and to varying degrees ambiguous. Our perceptual system needs to weight the sensory input with top-down information from perceptual memory to construct stable and reliable perceptual interpretations^22^. The constructive nature of perception^23,24^can be easily demonstrated with ambiguous figures, like the famous Necker Cube^25^ (Fig. 1, first row, right). During its prolonged observation our perception alternates between two about equally possible 3D interpretations (Fig. 1, first row, central figures). A number of reviews summarize behavioral and physiological findings on ambiguous figures and perceptual multistability^26–30^.

Observing an unambiguous cube variant can bias the percept of the subsequently presented ambiguous Necker Cube either towards perceiving it in the same 3D orientation as the unambiguous precursor (priming effect, positive hysteresis effect) or in the opposite orientation (adaptation effect, negative hysteresis (e.g^31–33^).

A similar phenomenon can be observed at a more complex level of perceptual interpretation with the iconic representation of letters and number. Bruner and Minturn introduced an ambiguous Letter /Number icon which can either be perceived as the number “13” or alternatively as the letter “B”. Observing first an unambiguous icon version, representing the number “13” most probably primes the perception of the subsequently presented ambiguous icon towards perceiving again the number “13” (priming effect). Corresponding priming happens if the letter “B” is first observed^34,35^.

Such priming and adaptation effects can be described as influences from the perceptual short-term memory on the observer’s current percept and will in the following be labelled as “perceptual conditioning effects”. Moreover, the literature describes a variety of ways how the perceptual history – on different time scales – can influence our percept at the current moment. Examples are ultra-short-term memory effects^36,37^, positive and negative hysteresis effects^38–41^or serial dependence^42–44^.

## Aim of the study

The aim of the present study was to test a new experimental paradigm that may provide an objective measure of visual imagination abilities. It applies the perceptual conditioning effect (priming and adaptation) introduced above and is based on previous studies using ambiguous figures or binocular rivalry stimuli combining a conditioning paradigm with an imagination task^45–48^. Our paradigm contains a Real Condition to quantitatively assess the influence of an unambiguous conditioning stimulus on the perception of the subsequently presented ambiguous test stimulus. A second Imaginary Condition measured how much the participants’ perception of the ambiguous test stimulus is influenced by the previous pure imagination (instead of observation) of the unambiguous conditioning stimulus, while the stimulus itself is absent. We correlated the amount of Real Condition with the amount of Imaginary Condition. This was done for two different stimulus categories, lower-level ambiguous and unambiguous 3D cube stimuli and for a new higher-level ambiguous Letter /Number icon stimulus, inducing either the percept of the number “8” or the letter “S”, and unambiguous variants thereof^49^. We further correlated the amount of Real and Imaginary Condition with the results from the VVIQ questionnaire^21^. With this paradigm we tested the following hypotheses:

(1) If our participants realize visual imagination in a vivid manner, imagination of an unambiguous stimulus should have similar qualitative (priming or adaptation) and quantitative (amount of conditioning) conditioning effects on the subsequent ambiguous stimulus variant as the real observation of the unambiguous conditioning stimulus.

(2) Moreover, participants with high scores in the VVIQ questionnaire (i.e. low visual imagination abilities), should show a low degree of imaginary conditioning and vice versa.

## Method

### Participants

Twenty-one participants (7 females; 14 males) with the mean age of 27.57 (SD = 3.4) and normal or corrected-to-normal visual acuity participated in the experiment. All participants gave their informed written consent to participate and were naive as to the specific experimental question. The study was performed in accordance with the ethical standards laid down in the Declaration of Helsinki^50^ .

The number of participants was determined applying a power analysis based on a previous conditioning experiment from our lab^51^, using the ambiguous Necker cube and unambiguous cube variants.

The study was approved by the Ethics Committee of the Institute for Frontier Areas of Psychology and Mental Health, Germany (IGPP_2022_10).

## Experimental setup

The experiments were conducted on a 27-inch computer monitor with a resolution of 1920 × 1080 pixels and a refresh rate of 60 Hz in a dark room. The distance between computer and the participant was approximately 114 cm. Responses were recorded using a Logitech response pad, and the experimental session was managed using PsychoPy software running on an Intel Core i7-9700 K processor operating at 3.6 GHz and 32 GB RAM.

### Stimuli

As stimuli we used the perceptually ambiguous Necker Cube and an ambiguous Letter /Number stimulus. For each of the two ambiguous stimuli, we used two unambiguous stimulus variants that corresponded to the two perceptual interpretations of the respective ambiguous stimulus as conditioning stimuli (see Fig. 1). The stimuli had a size of 10° x 10° visual angle for Necker Cube and 10° x 5° visual angle for Letter/Number stimuli. They were presented in white (mean luminance of 40 cd/m^2^; for the unambiguous lattices we calculated the average luminance of the four outer corners) on a dark grey background (0.01 cd/m^2^).

## Experimental procedure

**Fig. 2:**
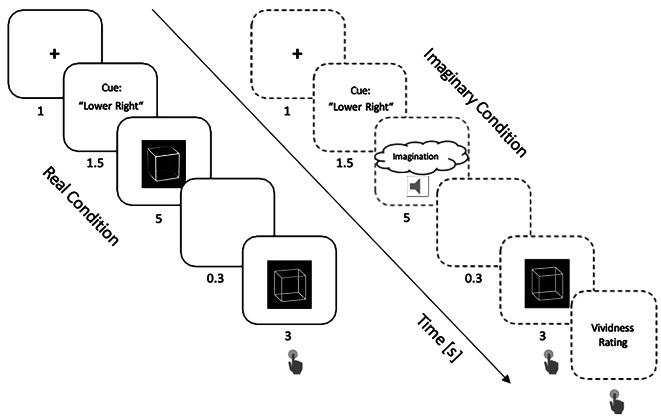
Experimental Paradigm. Each experimental trial from the both Real Condition and Imaginary Condition started with a red fixation cross for 1 s. For the next 1.5 s, a text cue indicated the unambiguous stimulus variant that was presented next (Real Condition) or that had to be imagined (Imaginary Condition). “Lower Right” / “Upper Left” were text cues for the cube experiment, indicating that the unambiguous cube variant that would be presented next has its front side pointing to the lower right / upper left. The announced unambiguous conditioning stimulus was presented for 5 s, followed by an inter-stimulus-interval (ISI) of 0.3 s. Finally, the ambiguous test stimulus was presented for 3 s and participants indicated their percept of it by pressing one of two possible keys. In the Imaginary Condition, the procedure was the same except, instead of showing the unambiguous conditioning stimulus, participants were instructed to imagine it. An audio tone, shown by an audio icon in the figure, indicated the end of the 5-second Imaginary Condition period. Moreover, after offset of the subsequent test stimulus participants had to rate the vividness of their imagination. The parts where participants had to respond are indicated by a response icon.

**Fig. 3:**
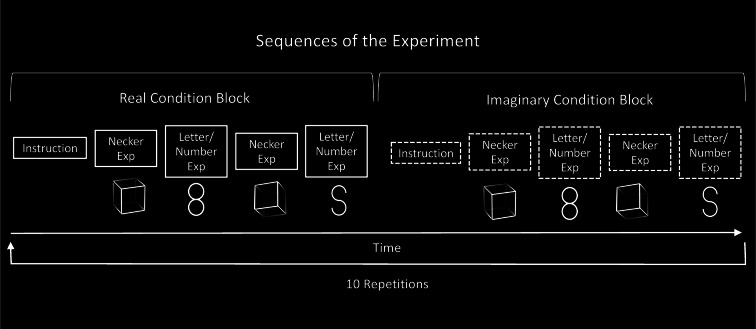
Sequence of the Experiment. The four different Real Condition trials (continuous frames and label on the left) were executed in a block followed by a block of Imaginary Condition trials (dashed frames and label on the right). This sequence of a Real Condition block, followed by an Imaginary Condition block was repeated 10 times. Each experimental block started with an instruction text, announcing the beginning of a Real Condition block or an Imaginary Condition block. The sequence of trials within a block was identical across blocks.

Upon arrival, participants read and filled out the “Vividness of Visual Imagery Questionnaire” (VVIQ) after reviewing the consent form. The study consisted of two experiments with two different stimulus types, Letter /Number and Necker Cube. Each experiment consisted of two experimental conditions, a Real Condition, and an Imaginary Condition (Fig. 2). The two experiments were executed in subsequent blocks as described in more detail in Fig. 3. Each block started with an instruction text, announcing the beginning of a Real Condition block or an Imaginary Condition block and the respective assignment of keys for participants’ responses. The instruction text was presented until participants pressed a key, that started the experimental block. Each block contained four trials as depicted in Fig. 3. The displayed order of trials was kept constant across repetitions and participants.

An experimental trial from the Real Condition started with showing a red fixation cross for 1 s. Next, a text cue occurred for 1.5 s, announcing the identity of the upcoming unambiguous conditioning stimulus. Subsequently, the conditioning stimulus was presented for 5 s, followed by an inter-stimulus interval (ISI) showing a blank screen for 0.3 s. Finally, the ambiguous test stimulus was presented for 3 s. Participants were instructed to observe the conditioning stimulus and report their percept of the subsequent test stimulus by pressing one of two keys on a keyboard.

A trial from the Imaginary Condition had the same design as a trial from the Real Condition with four exceptions:

(1) The text cues for the Imaginary Condition indicated not which stimulus will come next, but instead which stimulus had to be imagined during the following conditioning period, using the text labels introduced in Fig. 1.

(2) Instead of the unambiguous conditioning stimulus, a blank screen was presented and participants had to imagine the conditioning stimulus instead. Participants were allowed to facilitate imagination by closing their eyes.

(3) In order to indicate the end of the 5 s imagination period, an audio tone was presented.

(4) Participants were asked to rate the vividness of their imagination from 1 (no imagination at all) to 4 (perfectly clear imagination) after their perceptual response.

The Real Conditions were executed in a block followed by a block of Imaginary Conditions. This sequence of Real Condition and Imaginary Condition blocks was repeated 10 times (Fig. 3).

### Data analysis

We used Python 3.8 along with the SciPy library for data analysis. We calculated the probability of being primed, i.e., having perceived the test stimulus (Necker Cube and Letter /Number) in the same way as the previously perceived (Real Condition) or imagined (Imaginary Condition) conditioning stimulus. This was realized by dividing the number of prime percept responses by the total number of responses. A probability value of one thus means that all test stimuli had been perceived in the same way as the respective conditioning stimulus, indicating a maximal priming impact of the conditioning stimulus on the test stimulus. A value of 0.5 indicates chance level, i.e., the test stimulus had been perceived equally often in the same and in the opposite way as the respective conditioning stimulus. Furthermore, a value of zero means that all test stimuli had been perceived in the opposite way as the respective conditioning stimulus, indicating a maximal adaptive impact of the conditioning stimulus.

We tested for systematic priming and/or adaptation effects on the group level for each experiment and experimental condition separately by applying two-sided t-tests.

In order to test how much real conditioning is related to imaginary conditioning we correlated (Pearson correlation) the priming probability values of the Real Condition data with the respective values of the Imaginary Condition data. We did this separately for the two experiments.

For the correlation of the VVIQ questionnaire results with the conditioning effects we first normalized the conditioning effects. The idea behind this normalization was, that strong conditioning effects in both directions (either priming or adaptation) could be a sign of vivid imagination ability. Normalization was done in the following way: We first multiplied each conditioning value with factor 2 (range: 0 to + 2) and subtracted a value of 1 (range − 1 to + 1). We then took the absolute values (range: 0 to + 1). This transformation made a value of 1 representing both maximal adaptation and maximal priming. A value of 0, in contrast, represented chance level and thus neither adaptation nor priming influence of the preceding unambiguous stimulus on the ambiguous successor stimulus. Correlations were calculated using the Pearson correlation coefficient.

P-values were corrected using Bonferroni-Holm correction^52^.

## Results

### Priming and adaptation effects

**Fig. 4:**
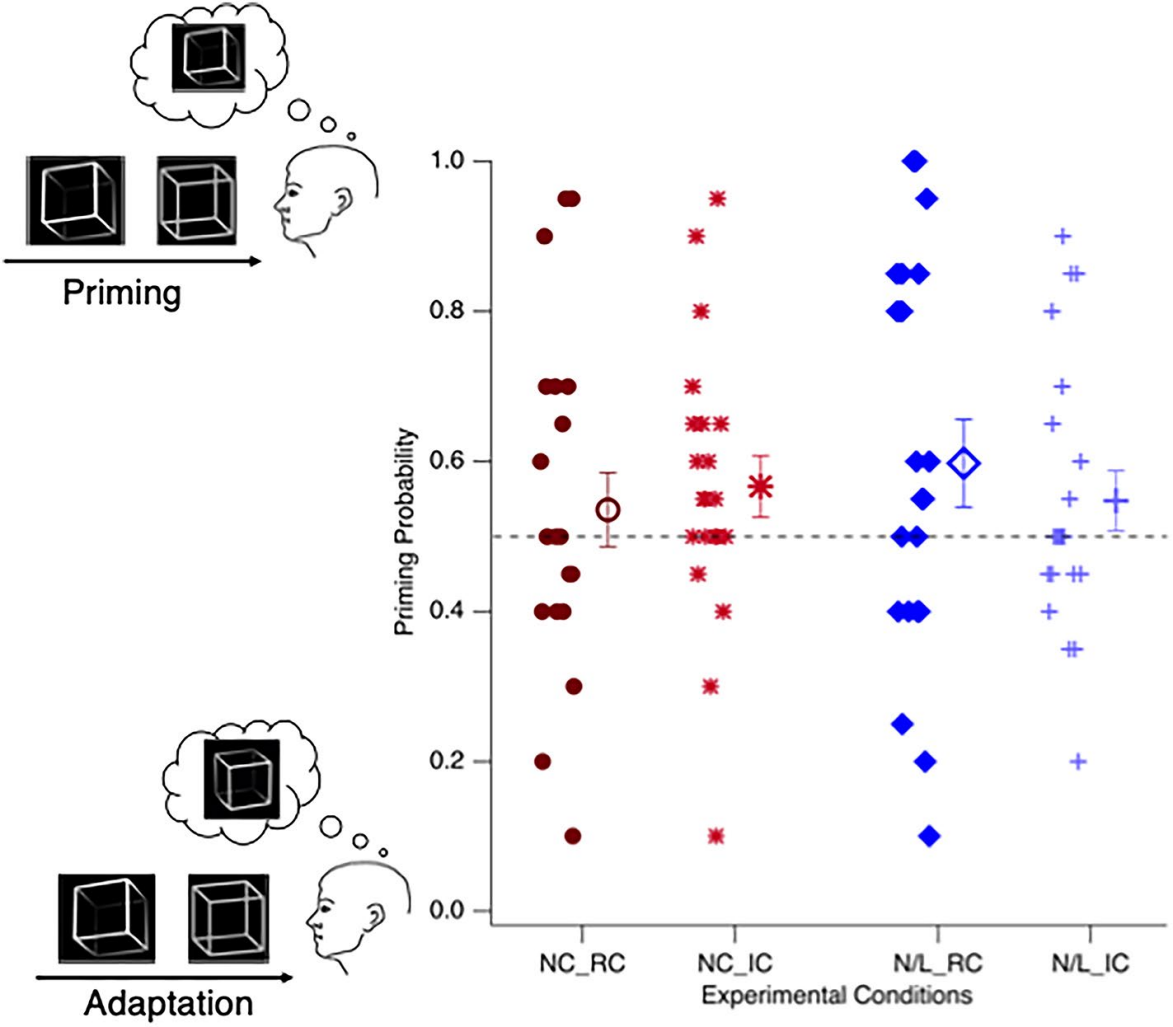
Results. The ordinate depicts the priming probability (the probability of having perceived the test stimulus in the same way as the preceding conditioning stimulus). A probability value of 1 means 100% priming (as indicated by the cartoon on the top left) and a probability value of 0 means 100% adaptation (as indicated by the cartoon on the bottom left). A probability value of 0.5 indicates chance level i.e. equal number of prime and adaptation percepts (indicated by the dashed horizontal line). The abscissa contains the two different conditions of the two experiments. Dark red circles / light red stars are individual data from the Real / Imaginary Conditions of the Cube Stimulus Experiment. Dark blue diamonds / light blue crosses reflect individual data from the Real / Imaginary Conditions of the Letter /Number Experiment. Larger and unfilled icons represent means ± SEM. NC_RC: Necker Cube Stimulus Experiment, Real Condition. NC_IC: Necker Cube Stimulus Experiment, Imaginary Condition. L/N _RC: Letter /Number Stimulus Experiment, Real Condition. L/N_IC: Letter /Number Stimulus Experiment, Imaginary Condition. The absence of a systematic deviation from chance level in one of the two possible vertical directions indicates no consistent conditioning effect on the group level.

Figure 4 depicts separately for each experiment and condition the priming probability values of each individual participant (filled icons) and the grand mean priming probability values across participants (larger and unfilled icons ± SEM).

Any systematic deviation of the probability values from 0.5 indicates effects in the respective directions in which the ambiguous stimuli are being seen, either in the same direction as the previously presented unambiguous Conditioning Stimuli (priming, values above 0.5) or in the opposite direction (adaptation, values below 0.5). The results neither indicate a strong priming nor a strong adaptation effect at the group level (Necker cube, Real Condition: *p* = 0.5; Necker cube, Imaginary Condition: *p* = 0.12; Letter/Number, Real Condition: *p* = 0.11; Letter/Number, Imaginary Condition: *p* = 0.25). In contrast, across experiments and conditions some single participants do show strong priming and others strong adaptation effects and again others show neither.

### Relations between real and imaginary priming

To investigate whether participants, who are strongly primed or adapted in the Real Condition, show the same effect in the Imaginary Condition, we calculated separately for the two experiments the correlations between the two experimental conditions.

**Fig. 5:**
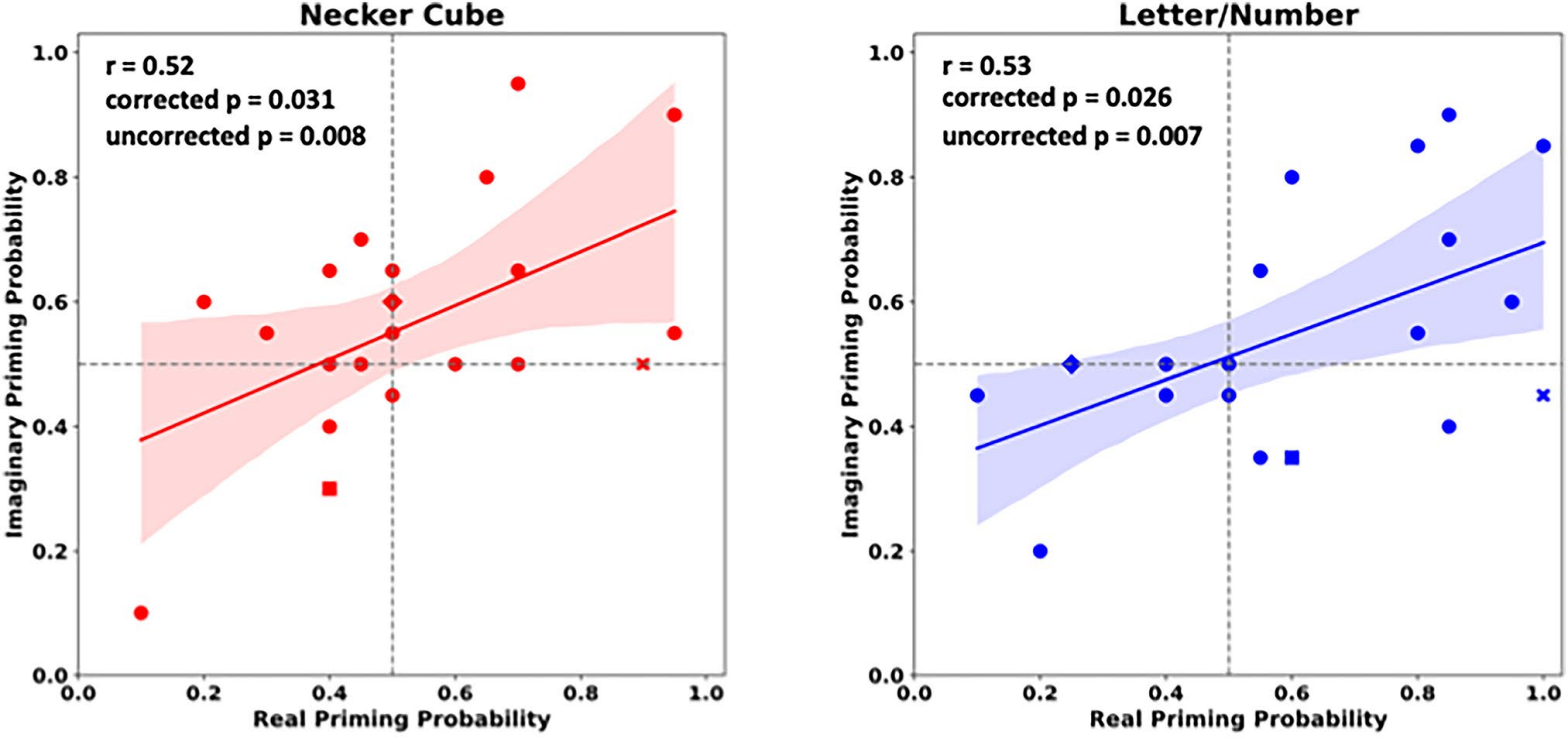
Correlations between Real and Imaginary Conditions. Scatter plots indicate the relation between Real and Imaginary Conditions separately for the Necker Cube Experiment (left, red data points) and the Letter /Number Experiment (right, blue data points). The abscissas indicate the real priming probabilities (i.e. the probabilities to perceive the test stimulus in the same way as the preceding conditioning stimulus). The ordinates indicate the imaginary priming probabilities (i.e. the probabilities to perceive the test stimulus in the same way as the previously imagined conditioning stimulus). The continuous lines show linear fits on the data. Note the data points of three self-defined aphantasics on each graph (diamond, square and cross).

The scatter plots in Fig. 5 indicate strong relations between Real and Imaginary Condition, both for the Letter /Number Experiment (*r* = 0.53; corrected *p* = 0.026; uncorrected: *p* = 0.007) and for the Necker Cube Experiment (*r* = 0.52, corrected *p* = 0.031; uncorrected *p* = 0.008).

### Relations between imaginary priming and results of the VVIQ

**Fig. 6:**
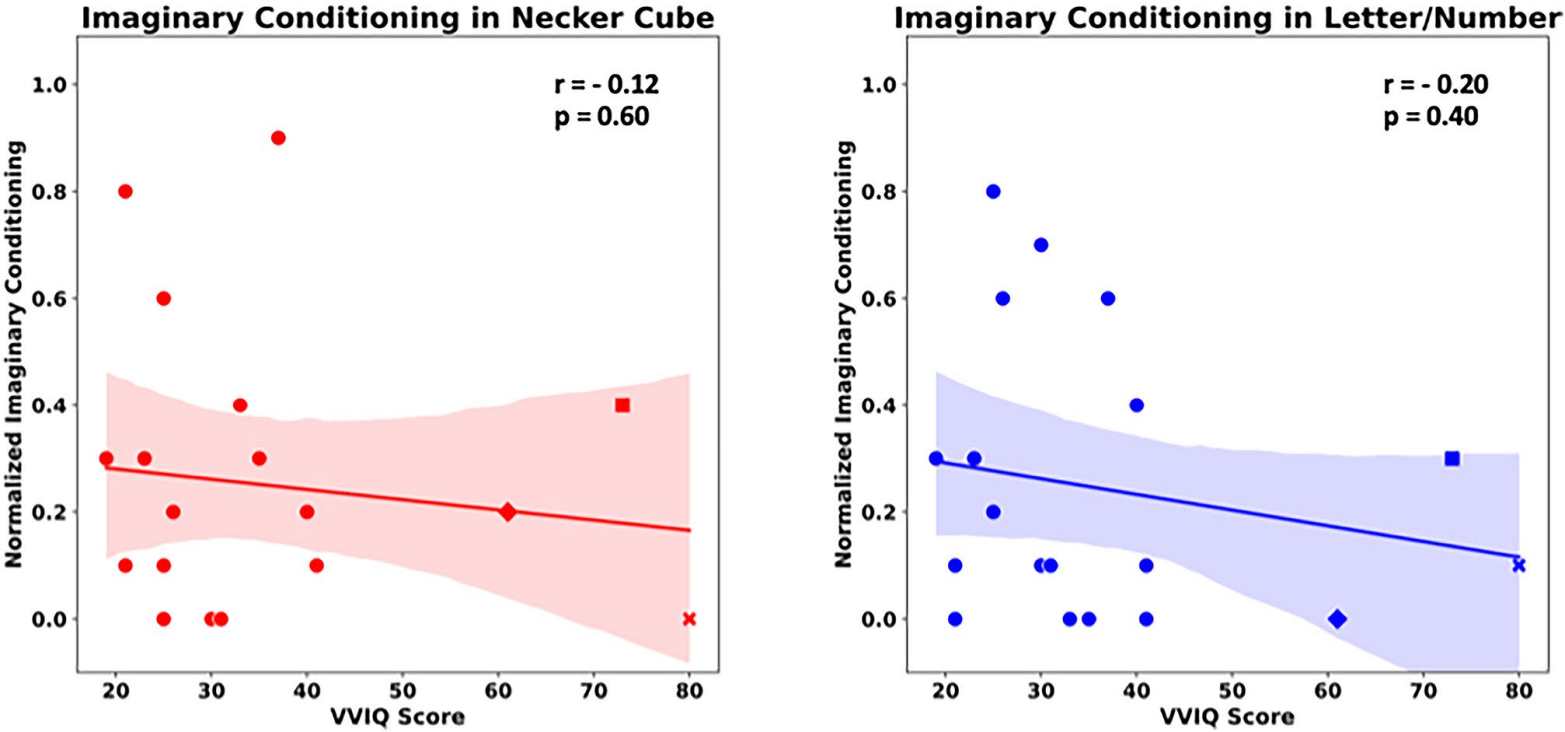
Correlations between imaginary conditioning effects and VVIQ results. Correlation plots show the relation between VVIQ questionnaire and imaginary conditioning separately for the Necker Cube Experiment (left) and the Letter /Number Experiment (right). The axes indicate the normalized conditioning probabilities (i.e., the probabilities to perceive the test stimulus either in the same way or opposite way as the preceding conditioning stimulus) against the VVIQ score for each participant. Note the data points of three self-defined aphantasics on the very right of each graph (diamond, square and cross). Note also, that large VVIQ values indicate weak imagination.

Figure 6 shows the correlation between participants’ scores on the visual imagination questionnaire (VVIQ) and the conditioning effects of visual imagery in the Imaginary Condition with the Necker Cube (left) and the Letter /Number (right) experiments. We did not find any significant correlation between the VVIQ and Necker Cube conditioning (*r* = − 0.12, *p* = 0.6) or Letter /Number stimulus conditioning (*r* = − 0.20, *p* = 0.4). However, a closer look at the graph provides an interesting qualitative result: Three participants in our sample regard themselves as aphantasics and reported this after the experiment to us. Interestingly, exactly these three participants formed a separate set of data points in Fig. 6 (three data points on the very right of each graph), reporting weak visual imagination abilities (questionnaire result) and showing relatively low conditioning effects.

## Discussion

Aim of the present study was to test a new experimental paradigm to quantify imagination abilities. For this, we used a conditioning paradigm with ambiguous stimuli and unambiguous stimulus variants from two different categories, i.e. Necker Cube and Letter/Number stimuli (Real Conditions). We extended the conditioning paradigm by a second experimental condition, where the conditioning stimulus wasn’t observed but instead had to be imagined (Imaginary Conditions). The basic underlying idea was to use the probabilities of imaginary conditioning as objective quantitative measures of participants imagination ability by correlating them to related probabilities of real conditioning. We further compared the probabilities of imaginary conditioning with VVIQ questionnaire scores as a subjective measure of the participants’ imagination ability.

We neither found systematic priming nor systematic adaptation effects on the group level. In contrast, some individual participants showed strong priming effects and some participants showed strong adaptation effects and again others showed neither. This pattern of results was observed across the Real Conditions of the two experiments and the Imaginary Conditions. Interestingly, our subsequent correlation analysis revealed that most of the participants with strong real priming or adaptation showed the same direction of conditioning and similar intensities in the Imaginary Conditions. This pattern of correlation results was consistent across Necker Cube and Letter/Number stimuli and is in line with results from a recent study by Poom and Matin from 2022 ^47^, who used ambiguous figure-ground stimuli.

We did not find a systematic relation between real or imaginary conditioning and VVIQ scores, which replicates again findings from Poom and Matin^47^. However, on a more qualitative level, we observed a small set of three participants with high scores on the VVIQ scale together with relatively low conditioning effects. These three participants identified themselves as aphantasics.

### Interpreting the absence of systematic adaptation and priming effects at the group level

A considerable number of experimental studies used ambiguous and unambiguous stimuli in selective conditioning paradigms, very similar to the present Real Conditions^31–33,38,47,53–59^. Most of those studies reported group adaptation and priming effects. It was particularly found that conditioning effects come with a retinotopic pattern. For example, no conditioning was found, if the conditioning stimulus was presented in one visual hemifield and the subsequent test stimulus in the other visual hemifield, or if conditioning and test stimuli differed substantially in size^33^. Moreover, both the quality (priming or adaptation) and the amount of conditioning was shown to be a function of the presentation duration of both the conditioning stimulus and the temporal gap between conditioning and subsequent test stimulus. More and larger priming effects were found with short conditioning periods and long gaps, whereas more and larger adaptation effects occurred with long conditioning periods and short gaps^31,32,60^. Possible dependent variables to assess conditioning effects included the initial percept of the ambiguous test stimulus immediately after its onset, the number of subsequent perceptual reversals across the duration of test stimulus observation and the total time of perceptual stability of the two different perceptual interpretations across the total time of test stimulus observation^31^. In the present study, we focused on the initial percept as dependent variable.

One interesting question is why we did not find any systematic classical priming nor systematic adaptation effects on the group level, like the previous studies? The reason for this may be that we used a fixed single value for the conditioning time (5 s) and for the gap duration between conditioning stimulus and test stimulus (0.3 s). In their seminal study, Toppino and Long^33^ tested conditioning times between 0 and 90 s and found priming effects for short conditioning times, and adaptation for long conditioning times. For conditioning times at around 1 s neither priming nor adaptation effects were found. The authors suggested that the two opposite conditioning effects neutralized each other in this case. According to their results, we should have found mainly adaptation effects with our 5-seconds conditioning time. Of course, the two studies differ in a number of experimental parameters, including the choice of stimuli. Another major difference is that in our study each Necker Cube trial was followed by a Letter/Number trial (see Fig. 3). In Toppino and Long’s study the conditioning trials were executed in succession without interruption. Whether this or the different stimuli explain the difference in results between their and our study has to be assessed in a follow-up study. In the present case, it is well possible that priming and adaptation time constants vary to a certain amount within a population and that the conditioning time we used supports priming in some participants, adaptation in others and a neutralization of the two opposite effects in again other participants, possibly resulting in a null effect on the group level.

Individual adaptation and priming time constants may be also interesting from the perspective of biological endophenotype effects. It may well be possible that such perceptual time constants are to some degree determined by genetic factors. Particularly, recent research on individual reversal rates (i.e. the number of perceptual reversals per time unit) during observation of ambiguous figures indicates the relevance of genetic factors^61–63^.

### About the relation between perception and imagination

The retinotopic character of the conditioning effects, as reported in the literature and described above, indicate mechanisms at lower levels of the processing hierarchy, where receptive fields are reasonably small. However, similar priming and adaptation effects have been reported for a variety of stimuli at very different levels along the perceptual hierarchy, from the perception of contrast^64^, motion^65,66^, line orientations^67^, up to the perception of the emotional content of faces^68^and even with numerosity^69^.

Mental imagination is the ability to activate perceptual representations from memory without any sensory input, inducing the experience of “seeing with the mind’s eye”^70^. It has been proposed that the brain uses almost the same “hardware” for visual imagination as for visual perception. In confirmation of this proposition, neuroimaging studies indicate similar neural representations when objects are imagined and when they are perceived (e.g^70^). A number of related findings further confirm this approach: Some behavioral studies reported that imagination content can particularly affect perception^71–73^. For example, imagination has been shown to affect visual detection thresholds^1^and performance on a visual acuity task^74^. Repeatedly visualizing the critical region of a visual bisection stimulus (visual spatial judgment) or a low-contrast Gabor pattern (contrast judgment) can enhance performance on subsequent perceptual tests^75^. Similarly, imagination of performing motor acts can improve performance on related tasks, probably by improving the connections between key areas of the motor cortex^76,77^. In addition, imagination can give rise to adaptation effects^78^ as much as a corresponding sensory stimulus.

The proposition of involvement of the visual-perceptual system during visual imagination together with its retinotopic organization fits well with the retinotopic pattern of priming and adaptation, as found in the literature, and also with our correlation analysis results. Let us assume that the intersection between neural structures that are active during perception of an ambiguous figure and during its imagination contains those structures that are responsible for priming and adaptation. Then it is reasonable that the imagination of an unambiguous stimulus variant has a highly similar effect on the subsequent ambiguous test stimulus as the real percept of the conditioning stimulus, with more or less the same time constants for priming and adaptation within individual observers. This is exactly what our correlation analysis indicates. Most of our participants who showed strong and those who showed weak priming or adaptation effects in the Real Conditions showed the same pattern in the Imaginary Conditions.

The present study is only on the level of a “proof of the principle” and the functional details need to be further clarified in future experiments, including measurements of the underlying neural mechanisms and comparing cerebral sources. Moreover, a considerable number of our participants showed priming probability values close to the chance level. Given that we only used one conditioning time, we cannot interpret these results unambiguously, as will be discussed below in more detail.

### Did we measure imagination strength or instead cognitive styles?

The correlation between Real and Imaginary Condition indicates that vivid visual imagination activates and therewith primes or adapts the perceptual system in the same way as visual perception does. Particularly, if our choice of conditioning time induced either a strong priming or a strong adaptation effect in the Real Condition, it induced the same type of conditioning (priming or adaptation) and with about the same intensity in the Imaginary Condition. Moreover, those participants who showed weak or no real conditioning effects produced comparably weak imaginary conditioning. According to this logic our results indicate that our paradigm may provide a good objective measure of imagination abilities and indicates at the same time a considerable overlap between neural resources underlying perception and imagination. However, we also applied the VVIQ questionnaire to measure the subjective vividness of imagination and correlated the resulting vividness scores with the behavioral conditioning variables. No significant relation between the vividness of imagination and the amount of imaginary conditioning was found.

A simple alternative to the above explanation may be the following: What we so far labelled as priming and adaptation effects – or the absence of both – in individual participants may reflect a kind of cognitive style rather than a lower-level effect in earlier visual/perceptual brain areas. Some participants may simply decide volitionally to perceive the ambiguous test stimulus either in the same or in the opposite direction as the preceding conditioning stimulus in the Real Conditions and in the same or in the opposite direction as the imagined stimulus in the Imaginary Condition. This strategy may alternatively explain our correlation results. It also can explain that we did not find any relation between our conditioning effects and the VVIQ results and it would not allow a substantial conclusion about the mechanisms underlying imagination.

Although we cannot rule out this explanation with our data, we will list two arguments against this explanation:

1. This cognitive style explanation necessitates the following additional postulate: Most of our participants need to apply the identical cognitive decision strategy consistently across stimulus types (Necker Cube, Letter/Number), the Real and Imaginary Conditions and across our 10 repetitions of each condition and conditioning stimulus variant, which is not very plausible.
2. As already summarized above, a number of conditioning studies with ambiguous test stimuli and unambiguous stimulus variants as conditioning stimuli have convincingly demonstrated a clear dependence of the direction (priming or adaptation) and strength of the conditioning effect on experimental parameters like spatial locations of and the inter-stimulus interval duration between conditioning and test stimulus. These dependencies clearly argue against the above introduced cognitive style explanation.

### Are the real and imaginary condition effects good predictors for visual imagination abilities?

One problem of the present study is that our participants with priming probability values close to chance level in both conditions, may be a mixture of different phenotypes, who cannot be separated based on the present data. Some participants may have high VVIQ scores but have conditioning probabilities close to chance level. They would have produced strong real and imaginary priming or adaptation effects with another choice of conditioning times. Others may a priori be unable to produce strong conditioning effects or any conditioning effects at all, independent of the conditioning time. Among those may be aphantasics, i.e. people who are unable to have a visual imagination. Keogh et al^46^ particularly focused on the phenomenon of aphantasia. They applied a similar conditioning paradigm as the present one to a group of predefined aphantasics and compared their results with a control group of non-aphantasics. In contrast to our choice of classical ambiguous figures as stimuli, they used binocular rivalry stimuli to induce conditioning effects^[e.g. 79^. Binocular rivalry occurs, when the two eyes see different stimuli at the same time^[e.g. 80^. Similarly to the classical ambiguous stimuli, perception alternates between the two eyes’ input. Keogh et al. found a correlation between the VVIQ scores and imaginary conditioning. However, their correlation is mainly based on the fact that their samples of aphantasics and controls form two clusters on the VVIQ scale. Our finding of no correlation between VVIQ scores and conditioning may be explained by the invisibility of potential conditioning effects, due to suboptimal conditioning times. The observation from our Fig. 6, however, indicating a separate set of our three aphantasic participants based on high VVIQ scores, is a qualitative confirmation of parts of Keogh et al.’s results. The discrepancy of results between the two studies can also be due to the different character of ambiguity of the classical ambiguous stimuli compared to binocular rivalry stimuli. In the case of the former, one and the same stimulus information enters both eyes. In the latter, the two eyes receive different inputs. There is a long-lasting debate of whether ambiguity effects with the two stimulus types can be compared^26,81,82^. In a follow-up study, it may be interesting to see whether observer with strong conditioning effects with ambiguous figures show similar effect sizes with binocular rivalry stimuli.

As a next step we need to assess optimal priming and conditioning times for each participant individually and execute the present experiment with individually optimized conditioning times. This may disentangle the potential mixture of subgroups with low and high conditioning results. We further need to test a separate group of aphantasics, to investigate whether our qualitative observations can be confirmed on a quantitative level.

### Outlook

It is well known that what we perceive at a present moment not only depends on what enters our eyes (bottom-up information), but to varying degrees also on what we have perceived in the past (top-down information)^13,67,83^. This is part of the constructive nature of perception. Our results, together with the findings from Keogh et al^46^, indicate that not only our previous percept (across different memory time scales) but also our recent visual imagination can have an influence on how we perceive the world around us at a present moment. The imagination literature teaches us that both real perception and imagination need the “perceptual brain” as the underlying hardware. Our correlation findings provide an easy and convincing way to behaviorally support this finding. Moreover, it may be possible to increase effect sizes and explanatory power, by using individually optimized conditioning time constants in the present experimental paradigm.

The latest results from aphantasia research, however, questions the generality of the relevance of the “perceptual brain” for imagination. Aphantasics may not be part of a continuum of visual imagination abilities, but instead represent a separate population with separate imaginary mechanisms. The present experimental paradigm is based on a similar idea as the binocular rivalry paradigm introduced by Keogh & Pearson^46^. With the Real Condition, it contains an important extension that may allow more precise statements about the potential mechanisms underlying “normal” visual imagination. Our paradigm may thus become a promising candidate for an objective measure of imagination abilities and to identify subgroups. Importantly, the choice of stimuli (ambiguous figures) and the simplicity of the task allow to assess imagination abilities in online studies and has thus the potential to acquire large amounts of data sets.

In 1995 Ishai and Sagi defined visual imagination as the activation of a perceptual representation in the absence of sensory input^1^. Hallucination is another type of perception without sensory input, which can occur in any sensory modality. People sense touch, without being touched, they hear voices in silence, or they see objects, forms or shapes, their environment cannot see or hear. Such percepts without sensory input are typically involuntary and occur often but not always in combination with diagnoses of psychiatric disorders. In such cases, the intrusive nature of the phenomenon can substantially decrease the quality of life.

There are also other cases of exceptional perceptual experiences without measurable sensory input. They also come sometimes with a severe psychological burden but without a clear clinical diagnostic and an unclear ontological status^84,85^.

One highly relevant question for basic research is now, whether all these cases of perception without sensory input, irrespective of the differences in real-life shaping, share some basic neuronal sources and/or functions. The present paradigm may become a good starting point for future research in this direction.

## Electronic supplementary material

Below is the link to the electronic supplementary material.

Supplementary Material 1

## Data Availability

All data on the measured variables indicating participants’ responding functions that support the findings of this study are included within this paper and its Supplementary Information files.
