# Supplementary material for: Visual imagination can influence visual perception – towards an experimental paradigm to measure imagination
Source: Sci Rep. 2024 Oct 18;14:24486. doi: 10.1038/s41598-024-74693-x (PMC11489727; doi:10.1038/s41598-024-74693-x)
Supplement: Supplementary file 1 — Supplementary Material 1 [file 41598_2024_74693_MOESM1_ESM.pdf]

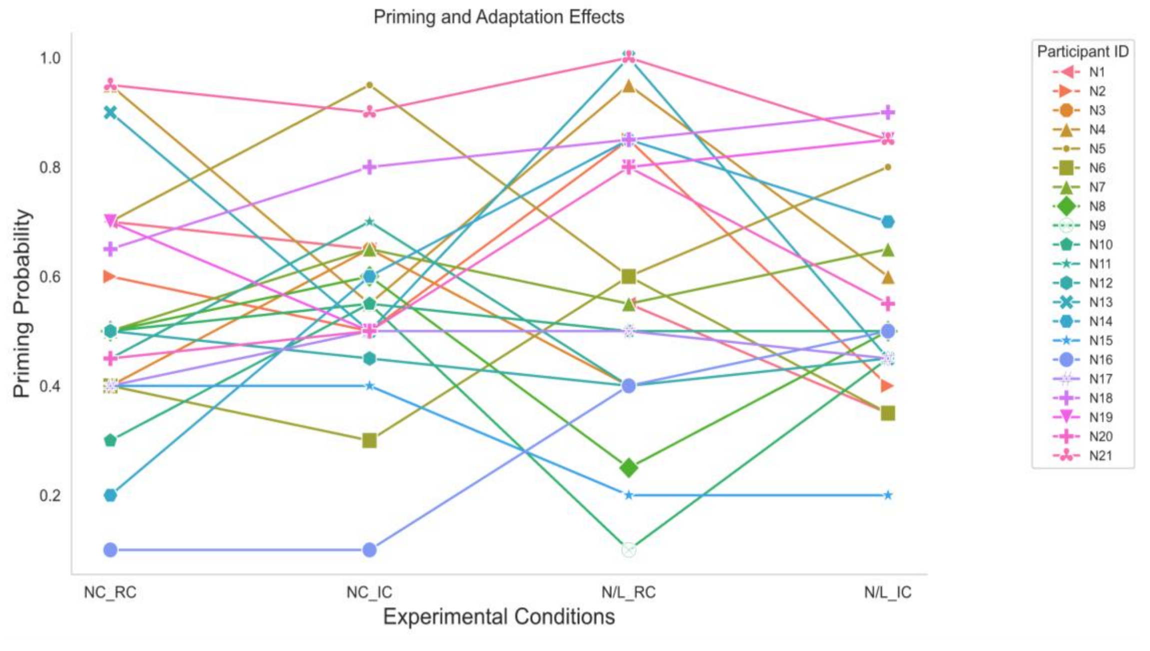

Supplementary Figure 1. Results. The ordinate depicts the priming probability (the probability of having perceived the Test Stimulus in the same way as the preceding Conditioning Stimulus). A probability value of 1 means 100% priming and a probability value of 0 means 100% adaptation. The abscissa contains the two different conditions of the two experiments. Each participant's data is represented by distinct markers and colors, and individual data points are connected by lines to show trends within participants from the Real / Imaginary Conditions of the Cube Stimulus Experiment as well as the Real / Imaginary Conditions of the Letter /Number Experiment.

NC_RC: Necker Cube Stimulus Experiment, Real Condition. NC_IC: Necker Cube Stimulus Experiment, Imaginary Condition. L/N _RC: Letter /Number Stimulus Experiment, Real Condition. L/N_IC: Letter /Number Stimulus Experiment, Imaginary Condition. The absence of a systematic deviation from chance level in one of the two possible vertical directions indicates no consistent conditioning effect on the group level. For both, the Necker Cube and Letter/ Number Stimuli, the majority of participants show comparable values for real and imaginary conditioning. This is also indicated by our correlation results.

Data

Supplementary Table 1:

ID	NeckerCube_RealCond	NeckerCube_ImagCond	Letter/Number_RealCond	Letter/Number_ImagCond
N1	0.70	0.65	0.55	0.35
N2	0.60	0.50	0.85	0.40
N3	0.40	0.65	0.40	0.50
N4	0.95	0.55	0.95	0.60
N5	0.70	0.95	0.60	0.80
N6	0.40	0.30	0.60	0.35
N7	0.50	0.65	0.55	0.65
N8	0.50	0.60	0.25	0.50
N9	0.50	0.55	0.10	0.45
N10	0.30	0.55	0.50	0.50
N11	0.45	0.70	0.40	0.50
N12	0.50	0.45	0.40	0.45
N13	0.90	0.50	1.0	0.45
N14	0.20	0.60	0.85	0.70
N15	0.40	0.40	0.2	0.2
N16	0.10	0.10	0.40	0.50
N17	0.40	0.50	0.50	0.45
N18	0.65	0.8	0.85	0.9
N19	0.70	0.50	0.80	0.85
N20	0.45	0.50	0.80	0.55
N21	0.95	0.9	1.0	0.85
